# Capacitive Sensor and Alternating Drive Mixing for Microfluidic Applications Using Micro Diaphragm Pumps

**DOI:** 10.3390/s22031273

**Published:** 2022-02-08

**Authors:** Thomas Thalhofer, Mauro Keck, Sebastian Kibler, Oliver Hayden

**Affiliations:** 1 Fraunhofer EMFT Research Institution for Microsystems and Solid State Technologies, Hansastrasse 27d, 80686 Munich, Germany; mauro.emilio.keck@emft.fraunhofer.de (M.K.); sebastian.kibler@emft.fraunhofer.de (S.K.); 2 Heinz-Nixdorf-Chair of Biomedical Electronics, TranslaTUM, Department of Electrical and Computer Engineering, TU Munich, Einsteinstrasse 25, 81675 Munich, Germany; oliver.hayden@tum.de

**Keywords:** micro pumps, capacitive sensing, microfluidic sensor, alternating drive mixing

## Abstract

Microfluidic systems are of paramount importance in various fields such as medicine, biology, and pharmacy. Despite the plethora of methods, accurate dosing and mixing of small doses of liquid reagents remain challenges for microfluidics. In this paper, we present a microfluidic device that uses two micro pumps and an alternating drive pattern to fill a microchannel. With a capacitive sensor system, we monitored the fluid process and controlled the micro pumps. In a first experiment, the system was set up to generate a 1:1 mixture between two fluids while using a range of fluid packet sizes from 0.25 to 2 µL and pumping frequencies from 50 to 100 Hz. In this parameter range, a dosing accuracy of 50.3 ± 0.9% was reached, validated by a gravimetric measurement. Other biased mixing ratios were tested as well and showed a deviation of 0.3 ± 0.3% from the targeted mixing ratio. In a second experiment, Trypan blue was used to study the mixing behavior of the system. Within one to two dosed packet sets, the two reagents were reliably mixed. The results are encouraging for future use of micro pumps and capacitive sensing in demanding microfluidic applications.

## 1. Introduction

Microfluidic devices are becoming more and more common in a variety of fields, including point-of-care diagnostics, pharmaceutical research, and biology [1]. A key challenge for every microfluidic device is the control of the fluid flow inside the system. Pure open-loop control of active fluidic devices is often inadequate because disturbances such as bubbles [2,3], phase boundaries [4], and changes in viscosity and temperature [5,6,7] can influence the flow. Therefore, many microfluidic systems use closed-loop systems based on flow sensors, which are based on mechanical cantilevers [8,9,10], thermal anemometers [11,12], or Coriolis force measurement [13,14].

Another commonly used method is capacitive fluid sensing. This sensing method uses the change in an electric field induced by moving fluids with different relative permittivities [15,16,17,18]. For example, the sensor system described in [19] uses the capacitance change induced by air bubbles injected into a fluid stream for continuous flow measurement. Another example for non-continuous flow is the dosing system developed by Zhang et al. [20], which uses a capacitive sensor electrode to measure the fluid amount dosed into a channel cavity.

Capacitive sensors have distinct advantages compared with other devices. Typically, the sensor structures are flat electrodes that are easy to manufacture and do not require direct fluid contact, making them reusable. The main disadvantage of capacitive sensors is the influence from environmental conditions causing drift and fluctuations. Therefore, capacitive sensors require a calibration for exact fluid volume measurements [17,21].

Another key challenge present in many microfluidic systems is the reliable mixing of reagents inside microfluidic channels [22]. Laminar flow, a characteristic for microfluidics, limits mixing through turbulence and convection. Therefore, either active mixers or elaborately designed channel structures are used to support the mixing process [23,24,25]. While many microfluidic mixing systems use continuous flow from different inlets, there exists another, less frequently used method: alternating drive mixing [23,26]. This mixing method alternatingly doses small fluid packets from two inlets into a channel, thus increasing turbulence and the contact area between the reagents. In order to perform alternating drive mixing, fast fluidic actuators such as micro pumps or micro valves are necessary.

In this paper, we present a novel microfluidic system intended for a three-stage radiolabeling process. The system uses two types of capacitive sensor electrodes for fluidic sensing and to control the mixing ratio: one electrode type for exact fluid position detection and a second type to measure relative fluid movement. Micro membrane pumps are used for fluid transportation, enabling alternating drive control. First, the system design and the fluidic process are described; then, in the experimental part, the dosing accuracy and mixing performance are evaluated. 

## 2. System Design 

**Figure 1:**
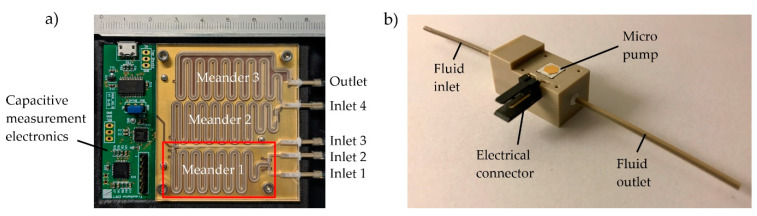
(**a**) Microfluidic channel structure and capacitive sensing electronics. The meander channels have a quadratic cross section of 1.5 × 1.5 mm², and the inlet supply channels have a rectangular cross section of 0.7 × 1.5 mm². The first of three meanders, which is examined in the following experiments, is marked with a red rectangle. (**b**) Fraunhofer EMFT silicon micro pump in a test housing adapter (pump dimensions: 5 mm × 5 mm × 1 mm).

The microfluidic system presented in this paper consists of a microfluidic channel system, a printed circuit board containing capacitance sensor electrodes, read-out electronics (Figure 1a), and a set of micro pumps for fluid transportation (Figure 1b). The sensor data are processed and converted into micro pump control signals using a python script on a control computer. The channel structure contains three large meander channels representing three successive dosing steps necessary for radiolabeling. Each meander has two entrances that are connected to either an external reagent reservoir or the previous meander. Micro pumps mounted between the reservoirs and the meander channels are used to intermittently dose small packets of reagents into the meanders. The fluid packet size ratio is monitored using capacitive sensing electrodes below the microchannel that can measure the progress of the gas–liquid interface as the channels fill. The micro pumps and reservoirs are connected to the fluidic chip using flexible tubings (LMT-55 1.02 × 2.72 mm, Pro Liquid GmbH, Überlingen, Germany).

### 2.1. Flow Process

The system is able to perform three successive dosing steps using three meander channels. Each meander uses its own set of electrodes to monitor the dosing process, but the algorithms and dosing principles are the same. For this reason, we focus this paper only on the first meander and its associated capacitive sensors.

**Figure 2:**
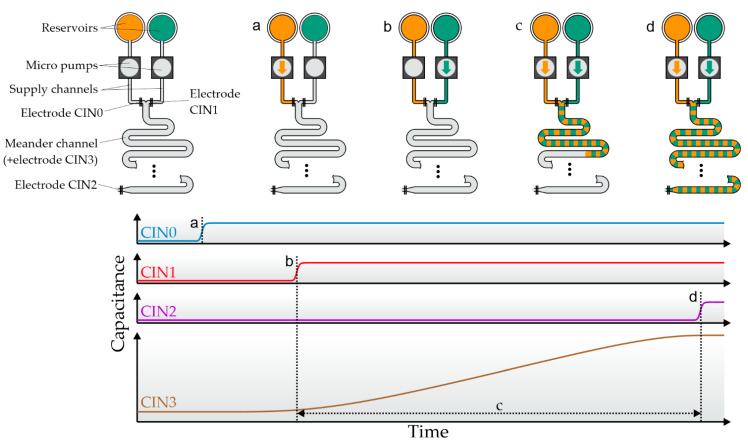
Schematic flow process and capacitance measurements during a complete dosing process in one meander. Four electrodes are used for fluid control, named CIN0 and CIN1 (at the end of the supply channels), CIN2 (at the end of the meander), and CIN3 (which covers the complete meander, not depicted). (**a**) Filling of the first supply channel. Filling is completed when digital electrode CIN0 detects fluid. (**b**) The second supply channel is full when CIN1 is triggered. (**c**) Alternating pumping. The capacitance of the main meander electrode is used to switch between the pumps. The capacitance gain caused by each pump is saved for later analysis. (**d**) The process finishes when CIN2 is triggered.

The dosing system uses capacitive sensor electrodes to detect the current fluid meniscus position and an algorithm to control the pumps. Figure 2 gives a graphical overview of the fluidic dosing process and the corresponding capacitive sensor data. First, the supply channels between the reservoirs and the meander are primed (Figure 2a,b). The end of this process step is marked by a capacitance change in the electrodes at the end of each supply channel. Second, the main meander is filled. One pump is activated and the capacitance of the electrode covering the meander is monitored. Once the measured capacitance reaches a desired threshold, the pump is deactivated, the capacitance gain is saved, and the second pump starts filling the mixing channel (Figure 2c). The second pump might use a different threshold if a mixing ratio other than 1:1 is desired. This process repeats until the electrode at the end of the meander is triggered (Figure 2d). Now the dosing step is complete, and the meander contains a mixture of two ingredients based on the defined mixing ratio. The absolute dosed volume is defined by the internal meander volume. The mixture can either be ejected (e.g., by using a third supply channel and pressurized air) or moved to a consecutive meander using one of the reagents as a propellant in order to perform the next process stage.

### 2.2. Micro Pumps

Two piezoelectric micro diaphragm pumps produced by Fraunhofer EMFT are used for fluid transportation. The silicon pump chips have the size 5 × 5 × 0.3 mm³ and a nominal pump chamber height of 20 µm. The nominal pump stroke volume is ~65 nl; however, the actual stroke volume may differ depending on the back pressure, actuation signal, and fluid viscosity. The pumps are driven using an alternating voltage of +90 V/−30 V. When negative voltage is applied, the piezo buckles outwards, effectively increasing the pump chamber volume. Applying positive voltage reverses this process and moves the piezo and the pump diaphragm towards the pump chamber bottom, ejecting fluid from the pump. The required actuation voltage is generated using a buck/boost converter circuit from a 5 V USB voltage source. The driver circuit is described elsewhere [19]. The pump rate can be set via the signal frequency and each voltage cycle induces a pump stroke.

The silicon pump chips are mounted on a modular housing adapter using a clamping mechanism. The adapter contains an upstream fritted filter, capillaries as fluidic fittings, and a 2.54 mm pin header for electrical connection. All wetted parts inside the pump are made of silicon. The housing, filter, and capillaries are made from PEEK.

### 2.3. Microchannels

The fluid structure is milled from a 3 mm acrylic sheet using a portal milling machine (Diadrive 2000, MUTRONIC GmbH, Rieden am Forggensee, GER) and single flute end mills (ø1 mm and ø0.6 mm, 10.000 RPM, feed rates 6.66 mm/s and 2.75 mm/s, feed depth 0.3 mm and 0.15 mm per pass, respectively). The channels have a depth of 1.5 mm and a width between 0.6 mm for supply channels and 1.5 mm for mixing meanders. The meander investigated in this paper by design has an internal volume of 495 µL. 

The open channels are sealed using 3M 9964 Transparent Microfluidic Diagnostic Tape (3M, Saint Paul, MI, USA). The tape contains a pressure-sensitive acrylic adhesive and can easily be applied onto the smooth, unmachined acrylic surface, while the hydrophobic properties prevent inadvertent fluid flow into the fluid channels by capillary forces. Fluid ports are added by drilling 1.8 mm boreholes into the side of the acrylic sheet and then inserting 1.6 mm PEEK capillaries (inner diameter: 1 mm) into said bores. The capillaries are glued into place and sealed radially using a biocompatible two-component epoxy adhesive (Loctite EA 9429). The excess capillary is cut to approximately 8 mm in length. Apart from the microchannels, the acrylic chip contains four screw holes and three bore holes for dowel pins, preventing lateral displacements between the acrylic channel structure and the electrodes on the printed circuit board.

### 2.4. Electronics and Hardware

An AD7142-1 capacitance-to-digital converter (CDC) integrated circuit (Analog Devices Inc., Boston, MA, USA) is used to measure the electrode capacitance. It operates by applying a 250 kHz rectangular signal to an excitation electrode and measuring the capacitance of a counter electrode using a 16-bit sigma-delta converter. The sensor chip is capable of measuring 14 capacitance inputs with a resolution of 61 aF and a working range of ±2 pF. Additionally, a bulk offset of up to ±20 pF can be added or subtracted from each input channel to increase the overall sensor range to ±22 pF. This bulk offset has a lower resolution of only 156 fF (20 pF @ 7 bit). Whenever the capacitance of an electrode is close to leaving the measurement range, the controller automatically stops the current process and sets a new capacitance offset to keep the electrode in range. The on-chip sequencer is programmed to continuously read and store the capacitance of all inputs that have electrodes connected to them, at a frequency of 30 Hz. 

**Figure 3:**
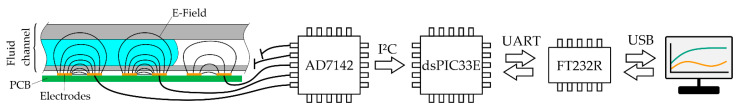
Main hardware components and communication protocols. The capacitance change induced by liquid filling the channel is detected by an AD7142 CDC. The data are continuously polled using a dsPIC33E microcontroller and sent to a control computer whenever requested by the control program at a rate of ~15 Hz.

The capacitance data gathered by the AD7142 are continuously polled by a dsPIC33E microcontroller (dsPIC33EP64MC202, Microchip Technology Inc., Chandler, AZ, USA) via a 200 kHz I²C interface. This microcontroller stores the latest data and serves as a communication interface between the capacitance sensor and the control computer. The microcontroller offers functions to read out capacitance and offset and provides functionality to automatically recalibrate the sensing electrodes (recalibration might be necessary on startup or if the capacitance exceeds the sensor range due to fluid filling the channel). The communication between the control computer and the microcontroller runs via a serial interface and string commands, using a FT232RL USB-to-UART IC (Future Technology Devices International Ltd., Glasgow, UK). The schematic data flow is visualized in Figure 3.

### 2.5. Electrodes and PCB

The electrodes are structured on a four-layer printed circuit board (PCB). The PCB is built around a 1.55 mm FR4 core, the internal and external conducting layers are separated using a 35 µm dielectric layer. The layer stack is as follows: (1) a top layer with sensing electrodes; (2) an internal layer that is empty (no copper); (3) an internal layer with a ground shielding plane; and (4) the bottom layer with tracks connecting electrodes and CDC.

The CDC used for the capacitance measurements uses a 250 kHz source applied to an excitation electrode and measures the system response of another measurement electrode to determine the capacitance. Each capacitance electrode therefore actually consists of two parts, an excitation part and a measurement part, which are separated by a gap. The excitation part of all electrodes is connected to the same driving source and each measurement part is connected to an individual CDC input pin. These inputs, and therefore also the associated electrodes, are referred to as Capacitance Input X (CINx), where X stands for the respective input pin index. In the following sections, the term “electrode” always represents a combination of an excitation part and a measurement part. Two different types of electrodes are used to sense and control the fluidic process, hereafter referred to as “digital” and “meander” electrodes, respectively.

The “digital” electrodes are located at the start and end of each channel section and are used to determine the exact position of the gas–liquid interface inside the microchannel (see CIN0, CIN1, and CIN2 in Figure 2). Each digital electrode consists of two 1.5 mm × 0.2 mm copper rectangles with 0.2 mm of separation between them as excitation and measurement electrodes. The digital electrodes are aligned perpendicularly to the fluid channel and the main flow direction in order to increase the spatial resolution and to minimize signal deviations due to lateral displacement between the PCB and the channel. A very similar electrode layout has been thoroughly investigated by Hoffmann [27]. The typical capacitance amplitude of these digital electrodes when fluid enters their channel section is about 3–20 fF, which corresponds to 50–300 sensor counts.

**Figure 4:**
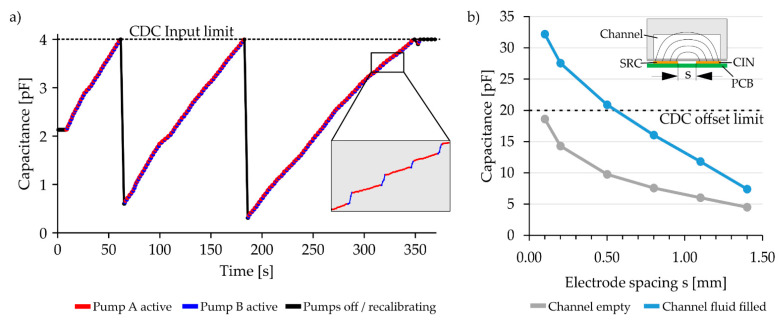
(**a**) Capacitance data measured by the “meander” electrode. The capacitance rises as fluid is pumped into the meander channel. The capacitance induced by each pump is used to determine when to switch the pumps. The insert shows exemplary data in more detail. The “blue” pump has a higher flow rate; therefore, the capacitance rises quicker when the blue pump is active. Recalibration steps are performed just before the meander capacitance reaches a value of 4 pF (=2^16^ counts). The recalibration moves the active sensor range by internally subtracting a capacitance offset and moving the measured capacitance back towards the bottom of the ±2 pF sensor range, thus increasing the high-resolution sensing range of the system. (**b**) Simulation data showing the capacitance of empty and full meander channels for variations in the electrode spacing s between the excitation electrode (SRC) and the measurement electrode (CIN). If s is smaller than 0.6 mm, then the capacitance of the associated meander electrode exceeds the CDC capacitance offset limit (dotted line) and hence the sensor range when the channel is filled with fluid. For the final system, an electrode spacing of s = 0.8 mm was chosen.

Over the complete length of a meander channel “meander” electrodes are positioned. Unlike the “digital” electrodes, which are only used to give a binary signal whether fluid is present or not, the “meander” electrodes continuously measure the capacitance change as the fluid slowly fills the meander channel. The capacitance change is used to determine when to switch between the pumps by keeping the capacitance change induced by each pump proportional to the desired mixing ratio. The process of switching between pumps based on the capacitance is shown in Figure 4a. In the following sections, only the first of the three meanders of the system is considered. This meander, and thus its associated “meander” electrode, has a total length of 220 mm. The excitation and measurement electrodes have a 0.35 mm width and are separated by a 0.8 mm gap.

The size of the gap separating the excitation and measurement electrodes directly influences the base capacitance and the capacitance stroke of the capacitance sensor: the smaller the gap, the larger the capacitance of the electrode and the more pronounced the capacitance changes as the meander is filled with fluid. Figure 4b shows the simulated capacitance of empty and full meander structures using different separation gaps (Simulations: EMpro 2019, Keysight Technologies, Santa Rosa, CA, USA). With a gap s < 0.6 mm, the capacitance exceeds the maximal CDC sensing range of 20 pF. In the opposite case with gaps that are too large, the capacitance change is minimized, reducing the measurement resolution.

For this study, a separation gap of s = 0.8 mm between the excitation and measurement electrodes was used. This yields to a capacitance of 7.5 pF for the empty channel and 16.0 pF for the water-filled channel. Since the capacitance change of 8.5 pF (= 16.0 pF − 7.5 pF) is larger than the 4 pF sensing range of the CDC, the CDC has to be recalibrated during the process. The recalibration process adjusts the input capacitance offset in order to move the electrode capacitance back into the sensing range of the CDC. These recalibration steps are visible as vertical black lines in Figure 4a.

## 3. Experiment: Dosing Accuracy

The total volume of a mixing step is always defined by the internal meander volume, but the reagent ratio inside the channel is controlled using the meander capacitance electrode. In this section, we investigate how accurately a desired reagent ratio can be set. This was done by running a dosing process and simultaneously monitoring the dosed volume of one pump using gravimetric measurements with a precision scale. The experiment was repeated for different package sizes (0.25 µL to 2 µL/packet), dosing speeds (pumping frequency: 50 Hz to 100 Hz), and mixing ratios (2.5:97.5 to 50:50) to observe the influence of these parameters on the dosing accuracy.

### 3.1. Preconsiderations: Real Meander Volume and Fluid Packet Size

The meander channel volume is an important parameter when investigating the dosing and mixing accuracy. By the CAD layout, the volume is supposed to be 495 µL, but due to manufacturing tolerances the real internal volume may vary. By gravimetric measurement using a WXTP-26 precision scale, the internal volume was measured to be 529.9 ± 1.0 µL.

**Figure 5:**
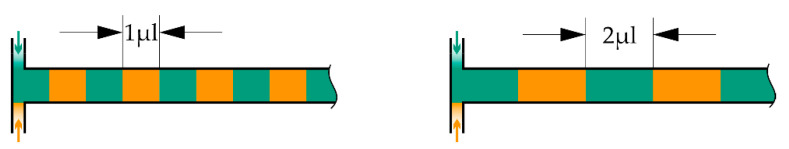
Visualization of different fluid packet sizes inside the microchannel. Of course, the zebra stripe pattern is for illustration purposes only and will not be present in reality due to mixing effects and diffusional transport.

The fluid packet size delivered by a pump before switching to the second pump (see Figure 5) can indirectly be set via the capacitance threshold. Using deionized water as liquid medium, the complete meander has a capacitance change of ~8.5 pF (the exact value might vary due to changes in permittivity inside the system, e.g., caused by temperature changes). Using a capacitance sensor with a 4 pF measurement range and 16-bit resolution, this is roughly equivalent to 140.000 sensor counts (8.5 pF/4 pF * 2^16^). Assuming a channel volume of 530 µL, this results in a resolution of 16 fF (264 counts) per µL. Therefore, if the system is, for example, set up to switch between the pumps using a threshold of 8 fF (132 counts), then the volume packets will be 0.5 µL in size. Since the channel has a 1.5 × 1.5 mm² cross section, its relative internal volume is 2.25 µL/mm; hence, a packet of 1 µL is approximately 0.44 mm in length.

### 3.2. Experimental Setup

Two micro pumps (pump “A” and pump “B”) were connected to the two inlet ports of the meander. Each pump was connected to an inlet reservoir containing particle-free, deionized water using flexible tubing. The reservoir of pump A was placed on a WXTP-26 precision scale (Mettler Toledo Inc., Columbus, OH, USA) with a 1 µg resolution. Both reservoirs were hydrostatically equalized to the meander to prevent leakage flow and additionally covered using polyethylene film (Duraseal, Diversified Biotech Inc., Dedham, MA, USA) to minimize condensation and evaporation. The pump outlet was connected to the dosing system inlet using a ~10 mm piece of flexible tubing, and the fluid channels were placed horizontally on a flat surface.

### 3.3. Experiment

First, the pumps, the fluid ports, and the supply channels were primed, using the digital electrodes as a reference, and the scale was nulled. Then, ten scale samples were recorded at a frequency of 1 Hz. Next, the channel was filled using the alternating dosing process described in Section 2. Both pumps were configured to alternatingly dose fluid packets of the same size (e.g., 0.5 µL), theoretically resulting in a mixture in which 50% of the fluid comes from pump A and 50% from pump B. During this process, the capacitance change induced by each pump was continuously logged. When the electrode at the end of the meander was triggered, the pumps were turned off automatically. Then, the meander fill time was logged and another ten scale measurements at 1 Hz were taken. Additionally, the capacitance change induced by each pump was recorded. After the data were gathered, the channel was dried using pressurized air and the next experiment iteration was started. This experiment was repeated n = 5 times for packet sizes of 0.25 µL, 0.5 µL, 0.75 µL, 1 µL, 1.5 µL, and 2 µL at a pumping frequency of 100 Hz, and again for 0.25 µL packet sizes at pumping frequencies of 100 Hz, 75 Hz, and 50 Hz. Then, using a pumping frequency of 100 Hz, a third parameter variation using four different mixing ratios was created, namely 2.5:97.5; 5:95; 10:90, and 25:75.

For each experiment iteration, the gravimetrically dosed volume of pump A was calculated by subtracting the last scale measurement before starting the pump from the first scale measurement after the pumps were stopped. Then, drift correction was performed, using the mean drift rate of the ten scale samples measured before and after the dosing process, multiplying this mean drift rate by the total dosing time and finally subtracting it from the dosed volume. The relative contribution of pump A was calculated by dividing the drift-corrected dosed volume by the total meander volume (529.9 µL). All experiments were carried out using the same fluid channels and capacitive sensing electronics.

### 3.4. Results

**Figure 6:**
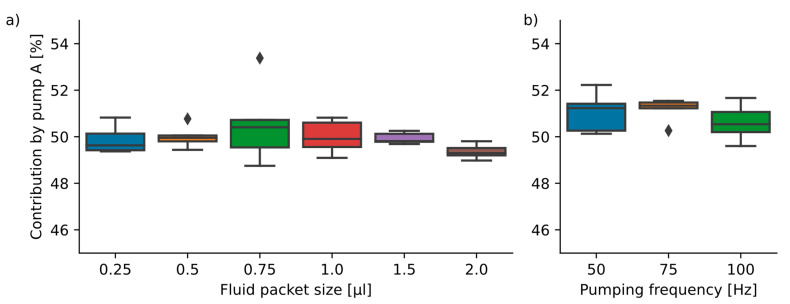
(**a**) Variation in packet size (*n* = 6 ∗ 5) at a 100 Hz actuation frequency. The mean and standard deviations are: 0.25 µL: 49.9 ± 0.6%; 0.50 µL: 50.0 ± 0.5%; 0.75 µL: 50.6 ± 1.8%; 1.00 µL: 50.0 ± 0.7%; 1.50 µL: 49.9 ± 0.2%; 2.00 µL: 49.4 ± 0.3%. (**b**) Dosing of 0.25 µL packets with a 50 Hz/75 Hz/100 Hz pump actuation frequency (*n* = 3 ∗ 5) yielded the results: 50 Hz: 51.1 ± 0.9%; 75 Hz: 51.2 ± 0.5%; 100 Hz: 50.6 ± 0.8%. No statistically significant differences between the groups were found.

Since both pumps A and B should contribute equally to the overall dosed volume in the packet size and pumping frequency variation, the expected contribution of pump A to the total dosed volume should be 50%. This is also reflected by the capacitive measurements, which indicated a mixture of 50.0 ± 0.1% (all results given as mean ± std) across all 45 experiments. The gravimetrically measured average mixing contribution of pump A across all experiment iterations was 50.3 ± 0.9%. Figure 6 shows a boxplot of the packet size variations (a) and the pumping frequency variations (b). The dosing time necessary to fill the complete meander continuously decreases from an average of 408 s (0.25 µL packets) to 247 s (1.5 µL packets).

**Figure 7:**
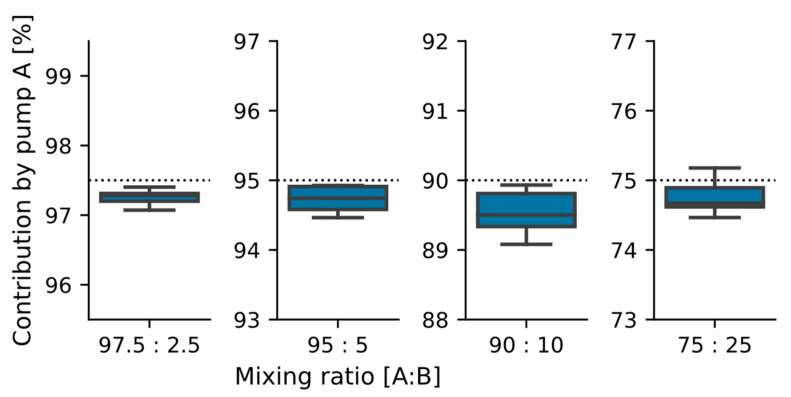
Dosing results for biased mixing ratios (*n* = 4 ∗ 5). The target dosing ratio for pump A is shown in the plots as a dotted line. The mean and standard deviations are: 97.5: 97.3 ± 0.1%; 95: 94.7 ± 0.2%; 90: 89.5 ± 0.3%; and 75: 75.8 ± 0.3%. The overall deviation from the targeted mixing rate for all *n* = 20 data points is 0.3 ± 0.3%.

The third varied parameter was the mixing ratio. The four biased mixing ratios other than 50:50 showed a deviation from the target mixing ratio of 0.3 ± 0.3%. Again, no statistically significant difference was found between the tested mixing ratios, indicating that the mixing ratio is freely selectable within the parameter range. The results are shown in Figure 7.

### 3.5. Discussion

In this first evaluation, a mean dosed volume ratio of 50.3 ± 0.9% was reached when performing a 50:50 mixture of two reagents, showing a very high accuracy of dosing. Neither the fluid packet size nor the pumping frequency had a significant influence on the dosing accuracy across the observed parameter range of 0.25 µL to 2 µL and 50 Hz to 100 Hz, respectively. A similar accuracy could be observed for biased mixing ratios, where a deviation in the respective targeted mixing ratio by 0.3 ± 0.3% was observed. Nevertheless, some outliers could be observed where the mixing accuracy was up to +3.4% higher than or dropped −1.2% below the targeted mixing ratio.

The source of these infrequently appearing inaccuracies is most likely air bubbles inside the micro pump, tubing, or channel system. The reason is as follows: when switching between the pumps, the pump is first turned off, and after a delay of 50 ms the capacitance gain detected by the meander electrode is recorded before the second pump starts pumping. If any fluid in the channel is still moving after this 50-ms period, then this fluid flow will be attributed to the second pump, even though it did not cause the flow.

Neglecting capillary and gravitational forces, there are two basic principles that can keep fluid moving in a microfluidic system after the driving pump has been stopped: inertance and fluidic capacitance. Due to the small amounts of liquid and the high internal friction in microfluidics, the inertance of the fluid column is typically not large enough to create significant flow. Fluidic capacitance is induced by air bubbles in the fluid path or by flexible fluidic structures. When the pump is active, it creates over-pressure in the fluidic system. This leads to an expansion of flexible structures and to a compression of air bubbles inside the fluid. When the pump is switched off, the flexible structures can relax and air bubbles can expand again, displacing fluid in the process. Since the flexible tubing used to connect the pumps to the channel is very short (30 mm) and relatively stiff, and since the lid foil covering the channel is backed up by the PCB, the contribution of these components to the overall fluidic capacitance is likely negligible compared with any air bubbles. For future devices, it would therefore be beneficial to introduce a bubble separator or a bubble trap in order to securely remove air bubbles from the fluid stream and to remove this potential source of error. Another method to cope with this problem may be to increase the off-time when switching between the pumps, but this would also increase the cycle times necessary to fill the meander.

In the second half of this experiment, multiple mixing ratios up to 97.5:2.5 were tested and showed accurate results. Even more extreme mixing ratios could be achieved, but the size difference of the fluidic packages has to be considered. The theoretical number of packets produced by each pump n depends on the minimal packet size $V_{P,\;min}$, the meander channel volume $V_{Meander}$, and the mixing ratio $r_{A}$:$r_{B}$. It is described by the following equation:

(1) $$n=\frac{V_{Meander}}{V_{P,\;min}*\frac{r_{A}\;+\;r_{B}}{\min\left(r_{A},\;r_{B}\right)}}$$

Assuming a minimal size of 0.25 µL for a single packet and a total meander channel volume of 530 µL, then the most biased mixing ratio tested in this paper leads to n = 53 packets delivered by each pump. Since the dosing algorithm uses each packet to correct previous dosing errors, using fewer than n = 20 packet pairs is not recommended and will likely lead to a larger dosing error.

## 4. Experiment: Mixing 

One major topic when trying to mix fluids in microfluidic channels is the quality of the mixing inside the channel. In this experiment, we used a colored liquid to investigate if and how the two fluids, that are pumped into the channel intermittently, are mixing inside the channel. In the previous section, we saw that we can choose the packet size. In this experiment, we investigated how different packet sizes influence the mixing process, and if we can find a maximum packet size for sufficient mixing.

### 4.1. Preconsiderations: Diffusive Mixing

**Figure 8:**
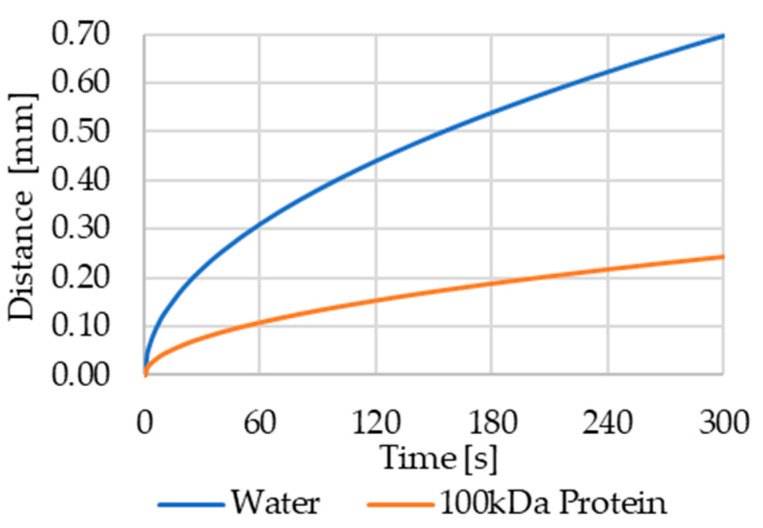
Average diffusion distance of water and a 100 kDa protein in aqueous solution. In 60 s, water molecules cover an average distance of 0.3 mm, while the protein will only cover 0.1 mm.

Microfluidics are typically in the laminar flow regime, which limits the contribution of turbulence to the mixing efficiency, leaving diffusion as one main mixing mechanism. Additionally, in microfluidics, two fluids usually enter a mixing channel segment simultaneously, but in the “alternating flow” process we use for this system, the fluids enter the channel in intermittent packages. Assuming we are dosing water as one reagent, and an aqueous solution of a 100 kDa protein as a second reagent, we can calculate the typical diffusion distance using the Stokes–Einstein equation:

(2) $$D=\frac{\langle x^{2}\rangle }{2t}=\frac{k_{B}T}{6\pi \eta r\;}$$

where D is the diffusion coefficient, $\langle x^{2}\rangle$ is the average diffusion distance, t is time, $k_{B}$ is Boltzmann’s constant, T is the temperature, η is the dynamic viscosity, and r is the molecule radius. Figure 8 gives a graphical representation of the mean diffusion distance over time for water molecules and a 100 kDa protein (D = $10^{-10}$ m²/s) in aqueous solutions.

In the previous experiment on dosing accuracy, the filling of the meander using a 1 µL packet size took on average 265 s. During this time, a 100 kDa protein covers an average diffusion distance of 0.23 mm. Since the channel’s cross section is 1.5 mm × 1.5 mm, the arithmetical length of a 1 µL packet is 0.44 mm. Due to the intermittent pumping, diffusion can happen from both ends of a fluid packet (each water packet is framed by protein packets and vice versa). Therefore, only considering diffusion, each water packet should be sufficiently mixed with the protein packages when it reaches the end of the channel. The meander bends, channel surface roughness, and non-stationary flow induced by the micro pumps should even increase the mixing efficiency.

### 4.2. Experimental Setup and Execution

Two micro pumps were connected to the two inlet ports of the meander. Each pump was also connected to an inlet reservoir, one containing only clear, deionized water, the second containing deionized water colored with Trypan blue. The reservoirs were hydrostatically equalized and covered using polyethylene film (Duraseal, Diversified Biotech Inc., Dedham, MA, USA) to minimize condensation and evaporation. Reservoirs, pumps, and meander channel inlets were connected with clear, flexible tubing. A video camera (Blackfly S U3-23S3C, Flir Systems, Wilsonville, OR, USA) was mounted about 25 cm above the meander channel and recorded the dosing process at 30 frames per second. The meander channels were placed on a flat surface, and the center line of the channel was approximately at the same level as the fluid in the reservoirs.

The pumps were actuated alternatingly, each injecting a fluid packet into the channel. The mixing of the colored and clear water was observed and evaluated visually using the captured video frames. The experiment was repeated for fluid packets with an approximate size of 1:1, 3:3, 10:10 and 1 µL:10 µL. After each experiment iteration, the channel structure was flushed with deionized water to remove dye residuals and dried using pressurized air.

### 4.3. Results and Discussion

**Figure 9:**
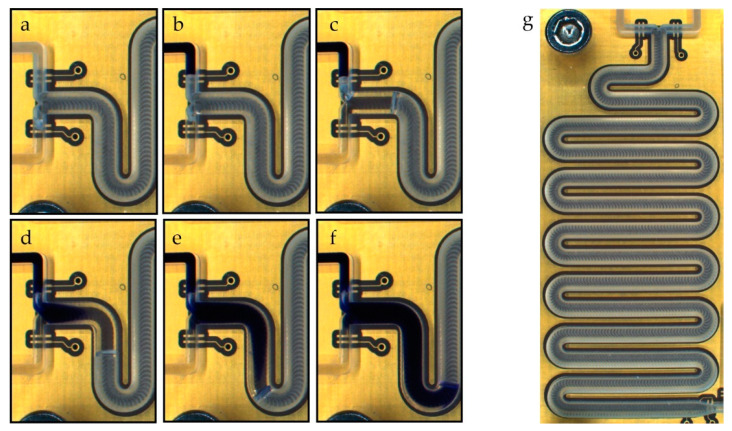
Alternate drive mixing of deionized water using Trypan blue as the dye. The image sequence shows the filling of the meander channels and two alternating dosing steps using a 10 µL packet size. (**a**) Water supply channel filled (left bottom channel). (**b**) Colored supply channel filled (left top channel). (**c**) First packet of pure water is dosed into the channel. (**d**,**e**): Colored water is dosed into the channel. (**f**) Result after dosing one packet of water and one packet of dyed water. (**g**) Image of the complete meander. The digital electrodes can be seen on each inlet and at the end of the meander. The golden meander electrode is slightly visible below the milled channel.

Complete mixing of the two fluids could be observed for all tested packet sizes from 1 µL to 10 µL. Regardless of the packet size, the fluid appears to be homogenously mixed after filling only 10 mm to 20 mm of the 220-mm-long channel. An image sequence illustrating the priming and mixing process for 10 µL fluid packets (the largest packet size tested) is shown in Figure 9. After filling the supply channels (Figure 9a,b), the process starts by injecting a packet of clear water into the meander channel (c), followed by a packet of dyed water (d–f). In snapshots (d) and (e), the parabolic flow profile typical for laminar flow can be seen as the dyed fluid moves towards the phase boundary.

The typical packet size used for the presented alternating drive process is significantly smaller than 10 µL. Smaller packets will mix even faster, firstly because of the diffusive mixing component described in the experiment preconsiderations, and secondly because smaller packets will be blended more by the fluid flow due to the higher surface-to-volume ratio.

## 5. Conclusions and Outlook

In this paper, we introduced a dosing system that uses capacitive sensor electrodes and micro pumps in order to generate a defined mixture of two reagents by applying an alternating drive process. No pre-calibration of the system is necessary by using “digital” electrodes to detect the fluid position at the beginning and at the end of each channel section, and “meander” electrodes to detect the relative movement of the fluid column inside the channel. While the total dosed volume is defined by the meander channel volume, the mixing ratio of the two fluid components in the channel can be freely chosen. In one experiment, we were able to demonstrate a high mixing ratio accuracy of 50.3 ± 0.9% when targeting a 50% mixture. Other, biased mixing ratios up to 97.5:2.5 were executed as well and showed an overall deviation of 0.3 ± 0.3% from the targeted mixing ratio. We were also able to show that neither packet size nor pumping speed nor mixing ratio significantly influence the dosing accuracy. In a second experiment, we found that the fluids were quickly homogenously mixed when alternatingly injected into the channel.

The capacitive sensing system presented in this paper has distinct advantages over other flow sensors used in microfluidics. Since the fluid sensing electrodes are not in direct contact with the fluid, they can be reused easily without the fear of contamination simply by replacing the fluid channel. By using an integrated capacitance-to-digital microchip, the system does not need bulky external hardware. The control software, which is currently running on an external computer, could be implemented directly on the microchip already included in the circuit in order to obtain a completely free-standing and encapsulable system. These properties make the presented capacitive sensing and dosing system an interesting candidate for application in nuclear medicine, where multiple systems could be integrated into a single hot cell to save lab space for the synthesis of radiopharmaceuticals and radiolabels. In the future, we want to apply the system to this environment and use it to automatically perform a multi-step synthesis process.

## Data Availability

Raw experimental results, pre-processed data, and videos of a mixing process in the fluidic system can be downloaded here: https://fordatis.fraunhofer.de/handle/fordatis/241 (accessed on 1 February 2022).
